# Gene expression alterations in brains of mice infected with three strains of scrapie

**DOI:** 10.1186/1471-2164-7-114

**Published:** 2006-05-16

**Authors:** Pamela J Skinner, Hayet Abbassi, Bruce Chesebro, Richard E Race, Cavan Reilly, Ashley T Haase

**Affiliations:** 1 Department of Veterinary and Biomedical Sciences, University of Minnesota, USA; 2 NIH Laboratory of Persistent Viral Diseases, Rocky Mountain Laboratories, Hamilton, Montana, USA; 3 Department of Biostatistics, University of Minnesota, USA; 4 Department of Microbiology, University of Minnesota, USA

## Abstract

**Background:**

Transmissible spongiform encephalopathies (TSEs) or prion diseases are fatal neurodegenerative disorders which occur in humans and various animal species. Examples include Creutzfeldt-Jakob disease (CJD) in humans, bovine spongiform encephalopathy (BSE) in cattle, chronic wasting disease (CWD) in deer and elk, and scrapie in sheep, and experimental mice. To gain insights into TSE pathogenesis, we made and used cDNA microarrays to identify disease-associated alterations in gene expression. Brain gene expression in scrapie-infected mice was compared to mock-infected mice at pre-symptomatic and symptomatic time points. Three strains of mouse scrapie that show striking differences in neuropathology were studied: ME7, 22L, and Chandler/RML.

**Results:**

In symptomatic mice, over 400 significant gene expression alterations were identified. In contrast, only 22 genes showed significant alteration in the pre-symptomatic animals. We also identified genes that showed significant differences in alterations in gene expression between strains. Genes identified in this study encode proteins that are involved in many cellular processes including protein folding, endosome/lysosome function, immunity, synapse function, metal ion binding, calcium regulation and cytoskeletal function.

**Conclusion:**

These studies shed light on the complex molecular events that occur during prion disease, and identify genes whose further study may yield new insights into strain specific neuropathogenesis and ante-mortem tests for TSEs.

## Background

Transmissible spongiform encephalopathies (TSEs) or prion diseases are fatal neurodegenerative disorders which occur in humans and various animal species. Examples include Creutzfeldt-Jakob disease (CJD) in humans, bovine spongiform encephalopathy (BSE) in cattle, chronic wasting disease (CWD) in deer and elk, and scrapie in sheep, goats and experimental mice [1]. TSEs are characterized by vacuolation of the neuropil, neuron loss, activation of astrocytes and microglia, and in some situations, deposition of amyloid fibrils [2,3]. While the misfolded partially protease resistant isoform of endogenous prion protein, also known as PrP^sc ^or PrP-res [4,5], is thought to play a critical role in these neuropathological changes, the molecular mechanisms underlying the neuropathology are far from clear [6,7].

With the goal of gaining insight into the basis of vacuolization and other neuropathological changes we undertook microarray studies in three mouse scrapie strains which differ in the neuropathological changes they induce. Here, we report the results of this investigation of mouse scrapie strains ME7, 22L, and RML-Chandler using cDNA microarrays. While some of these differentially-expressed genes have been described previously by others in related TSE systems [8-28], our studies extend gene profiling to include an important mouse scrapie strain, 22L [29], and in addition identify many new genes of potential importance in scrapie neuropathogenesis.

## Results and discussion

### Microarray analysis of scrapie-infected mice at two times post-infection

**Figure 1:**
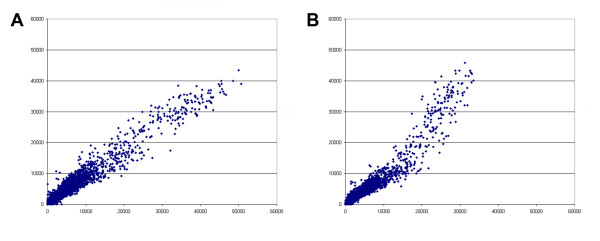
**Different scans of the same hybridized microarray show different results**. The representative hybridization results shown are from an ME7 infected mouse compared to a mock infected mouse. A) and B) show two scatter plots from two different scans of the same hybridized microarray. In each scatter plot, spot values from the mock infected animal are plotted on the X-axis and spots from scrapie infected animal are plotted on the Y-axis. Note that the scatter plots are different, and that in A) the spots more closely align with on the slope of 1 compared to the spots in B). The data from the scan presented in A) but not B) was used in these analyses.

Gene expression in six scrapie-infected mice was compared to gene expression in two mock-infected mice at 104 and 146 days post-infection (dpi) using cDNA microarrays. Of the six scrapie-infected mice, two were infected with strain ME7, two with strain 22L, and two with strain RML-Chandler. Day 104 was selected because it represents a time post-infection in which no clinical symptoms were observed and little neuropathology has been reported; and day 146 was selected because it was the time point empirically determined when the scrapie-infected mice showed clinical symptoms. In order to optimize data collected from microarrays hybridized with alternately fluorescently labeled cDNA from scrapie and mock-infected mice, we scanned each microarray at multiple laser settings ranging from relatively low power to high power. Resultant data collected for each spot on the hybridized microarrays was plotted on a graph, with values from scrapie infected samples on one axis, and values from mock-infected mice on the other axis. For each of the individual laser scans from each microarray, we found the data output when viewed as a scatter plot was highly variable, as exemplified in Figure 1. Only microarrays that showed a scatter plot of spot values from scrapie vs. mock with a slope of approximately one were considered for subsequent analysis. The reasoning for this selection is based on the assumption that most genes were not altered in the scrapie-infected animals, and thus for most genes spotted on the microarrays, fluorescently labeled cDNA from scrapie-infected mice would show hybridization equal to that of the fluorescently labeled cDNA from mock-infected mice. This selection process helped reduce artifacts from the microarray scanning process. As an additional means to optimize results, we also separately analyzed data from hybridized microarrays that were scanned with relatively high and relatively low laser power in order to best capture brightly and weakly stained spots, respectively. Further, each microarray contained two copies of each gene so that for each hybridization, we obtained results in duplicate. For each time point, the data from the high, low, and averaged data sets were separately analyzed using Significance Analysis of Microarrays (SAM) and produced unique data sets (indicated in Additional Data file 1, and Tables 1 and 2). These results demonstrate that laser intensities used to scan DNA microarrays can dramatically affect resultant data collected. Furthermore, these studies show that optimizing microarray scanning conditions allows for the identification of significant disease-associated alterations in gene expression using minimal numbers of mice.

**Table 1:** Genes with known function altered at 146 dpi with q ≤ 5% and ≥ 1.5 fold change

**Scan**	**ID**	**Name**	**SAM**	**q**	**ME7**	**22L**	**RML**	**Ave**	**Citations**
		**Endocytosis or Lysosomal function**							
L H A	AI838658	cathepsin B*	4	1	**1.4**	**1.7**	**1.3**	**1.5**	9 13
L H A	AI845967	cathepsin S*	15	1	**2.6**	**3.0**	**2.4**	**2.7**	9 12 11 14 18
L H A	AI838302	Cd63 antigen	5	1	**1.3**	**1.7**	**1.4**	**1.4**	
L	AI854206	hexosaminidase B*	4	1	**1.9**	**1.8**	**1.4**	**1.7**	9 11
H	AI841188	legumain	2	3	**1.0**	**1.5**	**1.3**	**1.3**	
H A	AI850263	lysosomal membrane glycoprotein 2	2	3	**1.0**	**1.6**	**1.2**	**1.2**	9
L A	AI845968	protective protein for beta-galactosidase	5	1	**1.2**	**1.5**	**1.5**	**1.4**	9
H A	AI852375	sortilin 1*	-3	1	**-1.2**	**-1.5**	**-1.1**	**-1.3**	
L H A	AI841326	transferrin*	4	1	**1.1**	**1.6**	**1.6**	**1.4**	9 22 20 23
		**Other proteases/protease inhibitors**							
L	SSHI-1h6	cathepsin Z	2	4	1.5	1.1	1.5	1.4	12 11
L H A	AI835498	cystatin C*	7	1	1.6	1.7	1.6	1.6	9 8
H A	AI842540	transmem. Pr. with EGF-like and 2 follistatin-like domains 1	-3	1	**-1.5**	**-1.1**	**-1.3**	**-1.3**	
		**Protein folding**							
L H A	AI848744	heat shock protein 1, beta	-3	1	**-1.0**	**-1.2**	**-1.5**	**-1.2**	
L A	AI844835	peptidylprolyl isomerase B	3	1	**-1.1**	**1.6**	**1.6**	**1.3**	
		**Immunity**							
L H A	AI848245	beta-2 microglobulin*	10	1	**2.6**	**2.9**	**2.2**	**2.6**	10 9 20 12 11
L H A	AI836786	complement component 1 q alpha*	6	1	**1.4**	**1.8**	**1.9**	**1.7**	1 8 12 11
L H A	AI854126	complement component 1, q beta*	4	1	**2.2**	**1.6**	**1.6**	**1.8**	1 8 12 11
H	AI853826	Fms interacting protein	-2	5	**-1.2**	**-1.7**	**-1.4**	**-1.4**	
H A	AI841111	lymphocyte antigen 6 complex, locus E*	2	3	**1.0**	**1.6**	**1.2**	**1.3**	
		**Calcium regulation**							
L	AI834839	calcium channel voltage-dependent T alpha1H	3	1	**1.3**	**1.4**	**1.6**	**1.4**	
L H A	AI835341	calmodulin 1	-4	1	-1.2	-1.3	-1.5	-1.3	
L H A	AI835663	calponin 3, acidic	3	1	**1.7**	**1.4**	**1.1**	**1.4**	9 11
L H A	AI853527	copine VIII	3	1	1.4	1.2	1.6	1.4	
H A	AI847702	inositol 1,4,5-triphosphate receptor 1	-3	1	-1.2	-1.5	-1.2	-1.3	
H A	AI839585	visinin-like 1	-3	2	**-1.5**	**-1.2**	**-1.3**	**-1.3**	
		**Cytoskeleton**							
L	AI841156	actin related protein 2/3 complex, subunit 1A	3	1	1.0	1.4	1.6	1.3	
H A	AI846176	capping protein (actin filament) muscle Z-line, beta	3	1	**1.3**	**2.3**	**1.1**	**1.5**	
L H A	AI836096	glial fibrillary acidic protein	6	1	**4.9**	**12**	**4.3**	**6.3**	11 8 41 25 26 27 19 17
L A	AI849905	neurofilament 3, medium	-3	1	**-1.3**	**-1.6**	**-1.3**	**-1.4**	10
L H A	AI845192	peripherin 1	4	1	**1.2**	**1.6**	**1.2**	**1.3**	
H	AI845991	RIKEN cDNA 4930488L10 gene	-2	4	**-1.6**	**1.0**	**-1.2**	**-1.2**	
L H A	AI845820	vimentin	6	1	**2.2**	**2.6**	**1.4**	**2.0**	10 9 8 19 11
		**Pumps or channels**							
L H A	AI841308	ATPase, Na+/K+ transporting, beta 1 polypeptide	-3	1	**-1.6**	**-1.3**	**-1.2**	**-1.4**	
L H A	AI836767	potassium inwardly-rectifying channel J 4	7	1	**1.6**	**1.8**	**2.1**	**1.8**	
L H A	AI839063	solute carrier family 25 (mitochondrial carrier), member 18	5	1	**1.4**	**1.5**	**1.2**	**1.4**	9
		**Lipid processing**							
L H A	AI840024	apolipoprotein D*	8	1	**1.5**	**2.6**	**2.3**	**2.1**	9 12 17
L H A	AI848248	apolipoprotein E*	8	1	**1.9**	**1.8**	**2.0**	**1.9**	9 18
		**Phosphatase or Kinase**							
H	AI852186	ectonucleotide pyrophosphatase/phosphodiesterase 5*	-2	5	**-1.1**	**-1.6**	**-1.4**	**-1.4**	
H A	AI841245	ethanolamine kinase 1	-2	5	**-1.5**	**-1.1**	**-1.1**	**-1.2**	
H	AI849290	mitogen activated protein kinase kinase 5	-2	4	**-1.3**	**-1.3**	**-1.6**	**-1.4**	
H A	AI842000	neurotrophic tyrosine kinase receptor type 2	3	2	**1.1**	**1.9**	**1.3**	**1.4**	9
H A	AI848471	protein tyrosine phosphatase, receptor type, F	-2	3	**-1.1**	**-1.3**	**-1.5**	**-1.3**	
H	AI854349	serum/glucocorticoid regulated kinase	2	4	**1.6**	**1.2**	**1.0**	**1.2**	9 12
L H A	AI854038	sorbin and SH3 domain containing 1	3	1	**1.6**	**1.3**	**1.0**	**1.3**	
		**Other enzymes**							
L H A	AI851848	aldehyde dehydrogenase family 1, subfamily A1	4	1	**1.2**	**1.7**	**1.5**	**1.5**	10 9
H A	AI838156	carbonic anhydrase 8	-2	3	**-1.3**	**-1.7**	**1.0**	**-1.3**	9
L H A	AI839962	malate dehydrogenase 1, NAD (soluble)	-3	1	**-1.1**	**-1.5**	**-1.1**	**-1.2**	9
		**Transcription or Translation**							
L	AI840211	A kinase (PRKA) anchor protein 8-like	2	4	**1.6**	**1.3**	**1.1**	**1.3**	
H	AI835606	eukaryotic translation elongation factor 1 alpha 2	-2	3	-1.0	-1.1	-1.5	-1.2	
H A	AI853820	eukaryotic translation initiation factor 5A	-3	1	**-1.5**	**-1.3**	**-1.4**	**-1.4**	
L	AI845998	GLI-Kruppel family member HKR2	2	5	**1.6**	**1.3**	**-1.0**	**1.3**	
L	AI847701	heterogeneous nuclear ribonucleoprotein D-like	-3	5	**-1.1**	**-1.8**	**-1.2**	**-1.3**	
L A	AI845667	mitochondrial ribosomal protein L9	2	5	**1.0**	**1.2**	**1.5**	**1.2**	
L A	AI838178	myotrophin	3	1	**-1.1**	**1.4**	**2.1**	**1.4**	
A	AI842636	phenylalanine-tRNA synthetase 1 (mitochondrial)	-2	4	**-1.5**	**-1.0**	**-1.2**	**-1.2**	
L A	AI851649	ribosomal protein L12	3	1	**1.1**	**1.2**	**1.5**	**1.2**	
H A	AI854670	RIKEN cDNA A730098D12 gene	-2	4	**-1.1**	**-1.1**	**-1.5**	**-1.2**	
L A	AI852411	SRY-box containing gene 9	3	1	**-1.0**	**1.2**	**1.5**	**1.2**	9
L A	AI838500	TSPY-like 2	3	1	1.0	1.5	1.7	1.4	
L	AI854130	TSPY-like 4	-3	5	**1.1**	**-1.3**	**-1.5**	**-1.2**	
		**Other**							
L H A	AI854515	CD9 antigen	5	1	**1.3**	**1.9**	**1.5**	**1.6**	12 11
L H A	AI836624	clusterin*	6	1	**1.6**	**1.6**	**1.6**	**1.6**	9 8 23 19 28
L H A	AI841459	diazepam binding inhibitor	5	1	**1.2**	**1.5**	**1.7**	**1.5**	9
L H A	AI854785	Endogenous retrovirus	3	1	**2.0**	**1.2**	**1.9**	**1.6**	21
H A	AI843767	endosulfine alpha	-2	5	**-1.4**	**-1.3**	**-1.5**	**-1.4**	
L H A	AI839644	ferritin light chain 1	5	1	1.4	1.2	1.6	1.4	
A	AI836826	glycoprotein 38*	2	4	**1.2**	**1.6**	**1.4**	**1.4**	
L H A	AI842053	lectin galactoside-binding sol. 3 binding prot.*	7	1	**1.7**	**2.4**	**1.8**	**1.9**	12 11
L H A	AI841372	N-myc downstream regulated gene 4	-4	1	**-1.1**	**-1.7**	**-1.3**	**-1.4**	
L	AI854649	oxysterol binding protein	2	4	**1.5**	**1.5**	**1.1**	**1.4**	
L	AI837057	Septin 3 (Sept3), mRNA	-3	5	**1.0**	**-1.7**	**-1.4**	**-1.3**	
L	AI845319	SPARC-like 1	3	1	**1.0**	**1.3**	**1.5**	**1.3**	
L H A	AI838871	synaptosomal-associated protein 25	-4	1	-1.5	-1.2	-1.3	-1.3	10 16
L H A	AI854259	thyroid hormone receptor associated protein 2	-3	1	**-1.1**	**-1.2**	**-1.5**	**-1.3**	
L H A	AI841166	Ywhah	-3	1	-1.2	-1.3	-1.5	-1.3	
H	AI854309	WD repeat domain, X-linked 1	-2	4	**-1.0**	**-1.1**	**-2.1**	**-1.3**	

SAM Score (T-statistic), q (q-value %), Ave (average fold change for all three strains)

Scan (Scan(s) that showed gene with significant alteration in scrapie brain)

L, H, and A (Low, High, or Average of Low and High powered scans)

Expression values shown for scan and clone that showed the highest SAM score.

ID (GenBank # for BMAP clones. IDs that begin with SSHI are from Rashmi Korke's mouse cDNA library)

Struckthrough BMAP accession numbers indicate that our sequencing results did not match the published sequence for that clone.

IDs with struckthrough BMAP accession numbers are listed with the gene name that reflects our sequencing results.

*Genes with an asterisk encode extracellular proteins.

**Table 2:** Genes that showed significant alterations (q ≤ 5%) in brains of scrapie-infected mice at 104 dpi

**Scan**	**Clone ID**	**Name**	**SAM Score(d)**	**q-value (%)**	**Ave. FC**
L	AI848245	beta-2 microglobulin*	2.0	4.2	**1.3**
L	AI836786	complement component 1 q alpha*	2.1	4.2	**1.3**
L	AI835314	cyclin-dependent kinase 5, regulatory subunit (p35)	1.9	4.2	**1.3**
L	AI851280	docking protein 4	1.8	4.2	**1.2**
L	AI854785	endogenous retrovirus, Intracisternal A particles	1.8	4.2	**1.2**
L	SSHI-2f6	endogenous retrovirus truncated gag	1.8	4.2	**1.4**
L	AI841933	eukaryotic translation initiation factor 3, subunit 2 (beta)	1.8	4.2	**1.2**
L	AI836096	glial fibrillary acidic protein	3.7	4.2	**1.9**
L	AI854206	hexosaminidase B*	2.0	4.2	**1.3**
L	AI848012	kinesin family member 3A	1.9	4.2	**1.3**
L	AI842053	lectin, galactoside-binding, soluble, 3 binding protein*	1.9	4.2	**1.2**
L	AI849127	unknown EST	2.0	4.2	**1.3**
L	AI854905	unknown EST	1.9	4.2	**1.3**
L	AI853719	unknown EST	1.8	4.2	**1.3**
L	SSHII-1h12	not sequenced	1.8	4.2	**1.2**
L	AI840066	Immunoglobulin superfamily, member 4B*	1.9	4.2	**1.3**
L	AI849299	Protein tyrosine phosphatase, receptor type Z, polypeptide 1	1.8	4.2	**1.2**
L	AI851594	RIKEN cDNA 1300010M03 gene	1.8	4.2	**1.2**
L	AI850724	RIKEN cDNA 2400003C14 gene	2.3	4.2	**1.3**
L	AI843193	RIKEN cDNA E130114P18 gene	2.2	4.2	**1.3**
L	AI840441	secretogranin III*	1.9	4.2	**1.2**
L	AI846048	RIKEN cDNA 2900073H19 gene	1.8	4.2	**1.3**

* Genes with an asterisk encode proteins that localize to extracellular space

Clone ID is GenBank # for BMAP clones. Clone IDs that begin with SSHI are from Rashmi Korke's mouse cDNA library

L stands for low scan

FC stands for fold change

Using this methodology, we identified over 400 genes that showed significant alterations in expression in the six scrapie infected mice compared to the two mock infected mice at 146 dpi (Additional File 1). For brevity, the subset of genes that have a known function and showed a fold change equal to or higher than 1.5 for one or more strain is presented in Table 1. Many of the identified genes have previously been described in related TSE model systems and are cited in Table 1 and Additional File 1.

Bioinformatic analyses, including literature searches, and the Source [46], the National Center for Biotechnology Information (NCBI), and the Database for Annotation, Visualization, and Integrated Discovery (DAVID) [30] data base searches, were used to identify the function(s) of each known gene identified in our study and group genes by their influence on various cellular processes. Many of the identified genes fell into more than one functional category. For simplicity these genes are presented under a single functional heading in Additional File 1 and Table 1. Dominant groups of genes identified include those that function in protein folding, endosome/lysosome function, immunity, the cytoskeleton, metal ion binding, calcium regulation and synapse function.

Because we investigated total brain gene expression, which amalgamates mRNA from all brain cell types including neurons, glial, and endothelial cells, we anticipate that gene alterations that occur in a single cell type or only in a particular region of the brain will show a low fold change in this analysis due to dilution by mRNA from surrounding cells or brain regions in which the particular gene alteration is not occurring. Thus, for microarray analyses involving complex tissues such as brain, low fold changes in gene expression may indicate large fold changes in a subset of cells or cells in a particular region of the tissue and should not be overlooked.

We've included the presentation of Additional File 1, which shows all significant alterations in gene expression identified in the scrapie-infected mice compared to mock-infected mice without a fold change filter applied (data with a fold change filter is presented in Table 1). When we filtered the data by fold change we lost the detection of several genes whose alteration may have significant impact on TSE disease pathogenesis or progression. For example several genes that function in protein folding showed a low but significant fold change and include: *Ahsa1, Dnajc1 Dnajc12, Dnajc4, Hspa12a, Hspca, Hspa4, Hspa8, Vbp, and Ppia*. Calmodulin 1 appeared as down regulated in the data set filter by fold change, but the unfiltered data set shows that calmodulin 2 and calmodulin 3 were also down modulated. Given this, we propose that it is important to consider genes that show significant changes in expression with albeit low fold change.

Surprisingly, only 22 genes were identified as significantly altered in the pre-symptomatic animals studied at 104 dpi. Six of the 22 genes were also identified as significantly altered in the symptomatic animals. These included glial fibrillary acidic protein (*GFAP*), complement component C1qa, lectin galactoside-binding soluble 3 binding protein, beta-2 microglobulin, hexosaminidase B, and an expressed sequence tag (EST) of unknown function. For each of these genes the fold change was substantially higher at 146 dpi compared to 104 dpi. Of the 22 genes, only one, *GFAP*, showed an average fold change of at least 1.5 (Table 2). The dramatic difference in numbers of gene expression alterations detected in the preclinical animals compared to the animals in late stage of the disease suggests that there is relatively little disruption in the brain until the late stages of the disease, at which point there is an abrupt massive disruption of multiple processes. Additional file 2 provides a visual representation of the microarray hybridization results for genes that showed significant alteration and at least 1.5 fold change in the scrapie infected mice.

### Gene expression alterations that varied among strains

**Figure 2:**
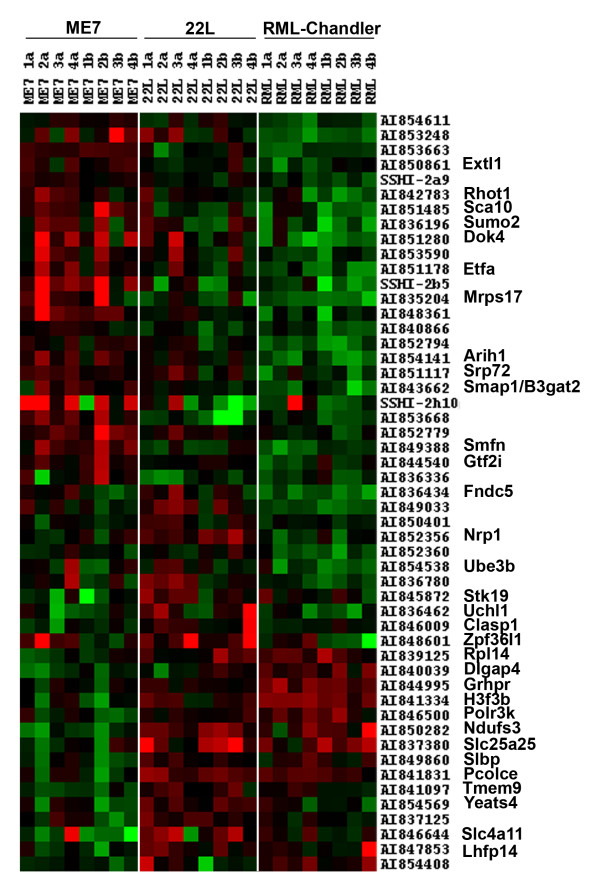
**Distinct patterns of alterations in gene expression produced by mouse scrapie strains ME7, 22L, and RML-Chandler**. In this Cluster and TreeView representation of genes that differed significantly between strains, sample information is listed across the top. Each scrapie-infected mouse was compared to each of two mock-infected mice resulting in four hybridizations per strain (1a–4b). Replicate results are indicated as 1b–4b. GenBank accession numbers and gene symbols are indicated. Note the distinct patterns of altered gene expression between the three strains.

Mice infected intracerebrally with scrapie strains ME7, 22L and RML-Chandler differ in incubation period and in neuropathology [29,31,32]. To evaluate whether there were alterations in gene expression that differed among scrapie strains, and, if so, thereby identify genes that may contribute to strain-specific neurodegenerative processes, gene expression profiles for each strain were compared by applying the Kruskal-Wallace test to the data. Because only two mice from each strain were compared to each of two mock infected mice, the smallest p-value obtainable using the Kruskal-Wallace test is 0.004. Yet, the results revealed 444 genes that showed significant differences among strains (p ≤ 0.05). The most significant 51 genes (p < .022) are shown in Figure 2. For each strain, the representative spots reveal a pattern that is quite distinct from the other two strains. The identified alterations in gene expression that differed between strains may represent genes that are involved with or perhaps even the basis for strain-specific degenerative processes in the brain. While additional studies with increased sample numbers are warranted to determine the reproducibility of these strain-specific findings, these results demonstrate that strains of scrapie used to inoculate mice can be distinguished based on the expression pattern produced by differentially expressed genes. These findings are supported by findings of Booth et al., who used a different set of mouse scrapie strains than the ones used in our studies and similarly found distinct strain-specific scrapie-associated gene expression patterns [10].

### Confirmation of DNA microarray results

As an internal control, our DNA microarrays contained several genes that have previously been shown to be altered in the brain during mouse scrapie infection including apolipoproteins E and D, glial fibrillary acidic protein (*GFAP*), *SNAP-25*, and Beta-2-microglobulin [18,20,24,26]. Negative control genes were also included in our arrays, including actin-b, and three plant genes: *CAB, RCA *and *rbcl *(Stratagene). Each of these control genes showed the expected scrapie-associated change or lack of change in gene expression. We also confirmed a subset of scrapie-associated alterations identified in our microarray studies using quantitative real time RT-PCR. The genes we evaluated included diazepam binding inhibitor, chemokine (C-X3-C) receptor 1, CD9 antigen, ATPase Na+/K+ transporting beta 1 polypeptide, cathepsin B, glial fibrillary acidic protein, and apolipoprotein E. Actin and Gapdh were included as negative controls. The results of the real-time PCR are presented in Table 3. We also sequenced nearly 400 of the cDNA clones that showed scrapie-associated alterations in our study. In instances where our sequencing results conflicted with BMAP sequences reported in GenBank, the GenBank accession number for the BMAP clone is struck through and the gene encoded by our sequencing is listed in additional file 1, Table 1, and Table 2.

**Table 3:** Real-time RT-PCR confirmation of microarray results

**Encoded protein/gene**	**Symbol**	**Microarray fold change**				RT-PCR fold change* (P value)			
			
		**ME7**	**22L**	**RML**	**Ave**.	**ME7**	**22L**	**RML**	**Ave**.
Diazepan binding inhibitor	Dbi	1.2	1.5	1.7	1.5	2.0 (0.021)	2.2 (0.009)	1.9 (0.026)	2.0 (0.001)
CD9 antigen	Cd9	1.3	1.9	1.5	1.6	2.9 (0.015)	4.2 (0.001)	3.7 (0.013)	3.6 (0.000)
CathepsinB	Ctsb	1.4	1.7	1.3	1.5	1.7 (0.019)	2.0 (0.003)	1.9 (0.009)	1.9 (0.011)
Chemokine (C-X3-C) receptor 1	Cx3cr1	1.2	1.2	1.4	1.3	1.8 (0.107)	3.2 (0.025)	2.0 (0.068)	2.4 (0.011)
Atpase, Na+/K+ transporting, beta 1	Atp1b	-1.6	-1.3	-1.2	-1.4	-1.3 (0.185)	-1.3 (0.195)	-1.3 (0.205)	-1.3 (0.02)
Glial fibrillary acidic protein	Gfap	4.9	11.7	4.3	6.3	11.6 (0.005)	14.6 (0.003)	12.3 (0.005)	12.8 (0.000)
Apolipoprotein E	Apoe	1.9	1.8	2.0	1.9	2.2 (0.005)	2.1 (0.013)	2.7 (0.086)	2.3 (0.002)
Glyceraldehydes-3-phosphate dehydrogenase	Gapdh	NA	NA	NA	NA	-1.1 (0.619)	-1.0 (0.798)	-1.2 (0.445)	-1.1 (0.393)
Actin, beta, cytoplasmic	Actb	1.1	1.2	1.0	1.1	1.0 (0.829)	1.3 (0.275)	1.1 (0.693)	1.1 (0.352)

NA, not available, Gapdh not represented on microarrays

*Fold change for each strain was calculated by dividing the average mRNA level from the 2 infected animals by the average of the relative mRNA level of the two control animals.

## Conclusion

We identified alterations in gene expression that occur in the brains of mice infected with scrapie strains ME7, 22L, and RML-Chandler at two times post infection. While it is difficult to compare our results with other previously performed gene expression studies in related TSE systems, because of the multiple differences in the methodologies used, the considerable overlap in the genes identified in our study and those identified in previous studies validate the importance of these alterations in gene expression in scrapie and suggest that these genes may generally play important roles in TSEs.

We identified for the first time novel scrapie-associated alterations in expression in numerous genes with known and as of yet unknown function. Many of the identified alterations in brain gene expression are likely important contributors to disease. In support of this notion, Klein et al., showed that complement gene knock out mice are resistant to intraperitoneal but not intracerebral inoculation of scrapie indicating that a single gene can be important to early events in peripheral transmission, propagation, and dissemination of scrapie in mice [33].

There are several genes identified in our studies that stand out as likely cofactors in TSE pathogenesis. For example, a large percentage of the genes we identified function in the endosome/lysosome system. These genes are of particular interest because the conversion of PrP^c ^to PrP^sc ^is thought to occur on the cell membrane or in the endosome/lysosome system and PrP^sc ^accumulates in endosomes and lysosomes of brain cells [34-37]. Over twenty percent of the scrapie-associated alterations identified in our study encode proteins that function in protein folding, protein degradation or localize to the endosomes/lysosomes system. We hypothesize that many of these genes are cofactors in prion protein misfolding and accumulation.

Other important scrapie-associated alterations identified in this study include genes that function in immunity, neuronal synapses, metal ion binding, calcium regulation, mitochondria and the cytoskeleton. Alterations in immunity genes are likely largely associated with microglial and astrocyte activation. Alterations in synapse genes may be important factors in the degeneration of neuronal dendrites. Many of the genes identified in this study bind metal ions, consistent with a role for disruption of metal ion homeostasis in scrapie neuropathogenesis. Alterations in genes that function in calcium homoeostasis and the mitochondria may be important factors in the degeneration of neurons, as maintenance of calcium homeostasis and energy production is critical for neuronal survival. Cytoskeleton gene alterations are likely reflective of migration of microglia, but may also be factors in other changes in cellular activity important to prion diseases such as vacuole formation in neurons.

C57Bl mice infected intracerebrally with scrapie strains ME7, 22L and RML-Chandler have similar time courses of disease and develop clinical symptoms within a week of each other at approximately 140 dpi. However, mice infected with these three strains of scrapie differ in the extent and localization of vacuolization and plaque formation. The intensity of vacuolation is highest in the anterior of the brain in mice infected with ME7, similar in various brain regions with RML-Chandler, and highest in the posterior of the brain with strain 22L. Strain 22L also shows a unique brain pathology in that it induces vacuolation in the cerebellar cortex and the vacuoles are larger in size compared to those induced by other strains of scrapie [29,31,32]. Strain specific alterations in gene expression detected in this study may indicate genes that contribute to the molecular mechanisms underlying strain specific neuropathology. However, from this study we can't distinguish alterations in gene expression between strains that are due to differences in neuropathology and those due to mice being at slightly differently stages of infection. In future experiments we hope to better understand the process of vacuolization by the analysis of regional expression of genes identified as strain-specific in our studies at multiple time-points post infection using in situ hybridization and immunohistochemical approaches.

Analysis of the cellular localization of the known genes showed that over 40 genes encode extracellular proteins (indicated with an asterisk in Table 1, Table 2 and additional file 1). If in future studies these proteins show TSE-specific differential accumulation in the cerebrospinal fluid or other accessible tissues, they could provide novel ante-mortem diagnostic markers for TSEs using simple antibody based or enzyme detection assays.

The studies reported here add to the handful of previously reported gene profiling studies of TSE pathogenesis using DNA microarrays. Our study included analysis of scrapie strain 22L which has not previously been reported in gene expression profiling studies, and implemented refined microarray analysis methodologies that allowed for identification of significant disease-associated alterations in gene expression using minimal numbers of mice. These studies are important because they shed light on the molecular mechanisms underlying TSE pathogenesis, identify potential surrogate markers for TSE diagnosis, and identify potential targets for drug treatment for TSEs. However, information gleaned from these studies represents just the tip of the iceberg of all of the genomic and other cellular and molecular changes that occur during TSEs. Additional studies are needed in order to unravel the rest of the mystery. The strain specific as well as strain non-specific alterations in gene expression identified in this study need to be evaluated using in situ hybridization and immunohistochemical approaches at multiple times post infection in order to determine the cell types in which alterations occur, the precise time post infection that they occur, and to determine whether specific TSE pathology including PrP^sc ^accumulation is associated with cellular populations showing alterations in gene expression. In addition, studies are needed to investigate gene expression changes in animals and humans infected with TSEs that have different genetic backgrounds including different alleles of PrP. Indeed Booth et al., found differences in alterations in gene expression in C57Bl/6 mice compared to VM mice infected with scrapie strain 22A [10]. Genomic studies in TSEs have thus far been confined to brain and need to be expanded to other tissues such as spleen and blood. Gene knock-out studies are needed to determine the extent to which each of the TSE-associated alterations in gene expression contributes to PrP^sc ^formation, and disease pathogenesis and progression. Cross-species studies are needed to determine alterations that are species specific and species non-specific. A comparison to other infectious and neurodegenerative diseases is needed to establish TSE-specific alterations from non-specific alterations. Studies are also needed to evaluate the use of genomic changes as surrogate markers for disease diagnosis, as well as the targeting genomic changes for potential drug therapies to treat TSEs. Thus, the scrapie-associated alterations in gene expression identified in the studies presented here and in those previously described have taken an important step in furthering our understanding the molecular mechanisms underlying TSE disease pathogenesis and disease progression. These discoveries provide a foundation for future studies that will take the next steps towards fully understanding TSEs, developing novel genomic based diagnostic assays for TSEs, and identifying targets for drug therapies to treat TSEs.

## Methods

### Scrapie infected mice

Three strains of mouse scrapie were examined: ME7, 22L, and RML-Chandler [29,38,39]. For each strain, four male C57BL/10 mice were inoculated intracerebrally with 50 ul of a 1% scrapie brain homogenate from clinically affected mice. The titer of each inocula was at least 2 × 10^8^infectious units/gram brain and exceeded the amount needed to induce disease in 100% of the animals [49]. Four control male mice were mock-inoculated with 1% homogenate of uninfected mouse brain. At 104 and 146 days post infection, two mice from each group were sacrificed. Scrapie infected mice sacrificed at 146 dpi exhibited clinical symptoms that included kyphosis, dull eyes, flattened stature, weight loss and ataxia.

### Preparation of microarrays

The Brain Molecular Anatomy Project (BMAP) cDNA library [40] containing 11,136 unique cDNA clones representing 8,700 genes was purchased from Research Genetics (now Invitrogen), and a largely uncharacterized 400 gene cDNA library encoding known and unknown mouse genes developed for an unrelated project was provided by Rashmi Korke and Wei-Shou Hu at the University of Minnesota for use in our microarrays. The cDNAs were amplified by PCR using primers designed from sequences of the cloning plasmid that flanked the inserted cDNA [41] and products analyzed on agarose gels. We were successful at amplifying over 90% of the genes. PCR products were purified using 96-well Millipore Multiscreen PCR cleanup kits, resuspended in 3 × SSC to a concentration of approximately 0.1 ug/g μl. DNAs were printed in duplicate onto lysine-coated slides using a BioRobotics Microgrid II (Genomics Solutions) DNA spotting robot. After printing, microarrays were baked and blocked excluding rehydration as described [42]. Slide quality was evaluated using Panomer 9-conjugated Alexa-594 dye (Molecular Probes, now Invitrogen).

### Microarray hybridization

Six scrapie-infected mice were compared to each of two mock-infected mice at both 104 and 146 days post infection (dpi). For each timepoint a total of 12 hybridizations was done with four hybridizations from each strain. Trizol reagent (Invitrogen) was used to extract total brain RNA from scrapie and mock-infected mice. RNA was reverse transcribed, amino-allyl coupled and labeled with Cy3 or Cy5 dyes (Molecular Probes, now Invitrogen) essentially as described [43], using 12.5 to 25 ug of RNA. In later experiments, 10–20% of the cDNA eluate was run on a 1% agarose gel and evaluated by spectrophotometry to check the quality and quantity of each reaction, and samples were adjusted if needed to yield equimolar and ideally at least 5 ug of labeled cDNA for experimental and control samples. For half of the hybridizations, Cy3-labeled cDNA from scrapie-infected mice and Cy5-labeled cDNA from mock-infected mice were combined and hybridized to microarrays in 40–60 ul of a solution that contained water, 50% formamide, 5 × SSC, 0.1% SDS, 20 ug polyA, 20 ug sheared salmon sperm DNA under 25 × 60 mm Lifterslips (Erie Scientific) in ArrayIt hybridization chambers (TeleChem) at 42°C O/N in the dark. Half of the hybridizations were done with the Cy3 and Cy-5 dyes swapped between experimental and control samples. Following hybridization, microarrays were briefly washed with 2 × SSC containing 0.1% SDS at 37°C, 2 × SSC at RT, 0.2 × SSC at RT, and then immediately spun dry.

### Array scanning

Hybridized microarray slides were scanned with ScanArray (PerkinElmer). High powered scans were collected such that spot intensities were as bright as possible to best detect weakly stained spots, and low powered scans were collected with only a small percentage of spots saturated to best collect brightly stained spots. QuantArray (Packard BioChip Technologies) was used to determine average pixel values for each spot from the scanned images and to identify poor quality spots which were not included in these analyses. Data was exported to Microsoft Excel and using the QuantArray QAreduce macro spot values were normalized based on total average spot intensity and background subtracted. To evaluate the quality of the data from each scanned microarray, a scatter plot was created by plotting mock verses scrapie-infected normalized and background subtracted spot values. Variation was observed in scatter plots from different scans of the same hybridization and scans that yielded scatter plots that predominantly centered on a slope of one were selected for our analyses. This selection process greatly improved the quality of the data that was ultimately analyzed.

### Statistical analyses and data mining

The first set of statistical analyses evaluated the results from 12 microarray hybridizations at each timepoint. The normalized and background-subtracted spot ratios from each scan were transferred to an excel worksheet. Duplicate spot ratios were moved to one row and linear spot ratios were converted to Log_2_. Data from the high scans and low scans were separately analyzed, as was data obtained from averaging the spot ratios from the low and the high scans. Significance Analysis of Microarrays (SAM) with false discovery rates (FDR) and q-values were used to identify genes that showed significant differential expression in scrapie-infected brains compared to mock-infected brains [44,45]. A One Class Response analysis was run with data input parameters kept at the default values. The results from SAM analysis include a SAM score and q-value. The SAM score is the T-statistic value. The q-value is the lowest false discovery rate at which a gene is called significant [45]. For example, a q-value of 1% indicates a gene has a 1% chance of being falsely identified as significant. The q-value is similar to a p-value and is the preferred measure here since the problem of multiple comparisons makes interpretation of individual p-values difficult. Fold change presented for each strain is the average scrapie:mock ratio of normalized and background subtracted microarray spot values. For ease of interpretation of the data presented, fold change values less than one were converted using the equation -1/spot ratio, e.g. values of 0.5 were converted to -2.

To identify genes that showed scrapie-associated alterations that were significantly different between strains, the Kruskal-Wallace test was performed on data from the 146 dpi time point [46]. As a means to verify the results from the Kruskal-Wallace test, a pairwise Wilcoxon test was performed on spot ratios from ME7 vs. 22L, ME7 vs. RML, and 22L vs. RML.

In addition, several data mining and visualization softwares were used including: GeneSpring (Silicon Genetics), Eisen's Cluster and Treeview [47], PathwayAssist (Stratagene), Medline, the National Center for Biotechnology Information (NCBI), and the Database for Annotation, Visualization, and Integrated Discovery [30]. BMAP gene annotations were updated using the Source [48].

### Quantitative real time RT-PCR

A Roche Light Cycler and Roche Light Cycler-RNA amplification kit-SYBR green I was used for quantitative real time RT-PCR reactions. Reactions were performed in 10 ul using 100 ng of DNAse treated total RNA. PCR primers were designed using MacVector software (Accelrys Inc.), and spanned introns. A stock of normal mouse brain RNA was made, treated with DNAse, purified and used with each RT-PCR to generate a standard curve, which was used to verify the efficiency of RT-PCR, and as a standard to normalize results from experimental samples. RNA from the same eight mice used for the microarray analysis at 146 dpi was used for this analysis. The relative mRNA levels were calculated using the 2^-ΔΔCT ^method using the equation 2^-(Sample CT-average of standard CT) ^with results from scrapie or mock infected RNAs as the sample, and results from normal mouse RNA amplified with same primers as the standard [49]. The fold change was calculated using the ratio of the average relative mRNA level from the scrapie samples/the average relative mRNA level from the mock-infected samples. PCR products were run on a 4% low melt agarose gel to verify the size of product. For each gene studied, a t-test was performed on the log of the relative mRNA levels of the target samples to verify the significance of the results. Similar to the microarray results, fold change values of less than 1 were converted using the equation -1/fold change, for ease of interpretation.

### Sequence verification

Plasmids were purified from BMAP and Rashmi Korke's bacterial stocks using Qiagen QIAprep spin miniprep kits and were sequenced at the UMN Advanced Genetics Analysis Center. The same primers used for PCR were used for sequencing. Blast2 analysis determined whether the sequence of cDNA clones used in our studies matched the reported BMAP GenBank sequences [50]. In instances where our sequencing results did not match the reported BMAP sequence, a BLASTn search was performed to identify homologous sequences.

## Abbreviations

transmissible spongiform encephalopathies (TSEs), Creutzfeldt-Jakob disease (CJD), bovine spongiform encephalopathy (BSE), chronic wasting disease (CWD), Brain Molecular Anatomy Project (BMAP), Significance analysis of microarrays (SAM).

## Authors' contributions

PS worked with AH and BC to develop the experimental plan, she also lead the production of cDNA microarrays, trained staff to make and use cDNA microarrays, performed the majority of the cDNA hybridizations described in this manuscript, did subsequent microarray and bioinformatics analyses, and drafted this manuscript; HA did five of the 24 microarray hybridizations described in this manuscript, performed the real time quantitative PCR, and helped with manuscript preparation; BC helped develop the experimental plan, authorized and oversaw scrapie infections, and help with manuscript preparation; RR did the scrapie infections and extracted the RNA used for these studies, and helped with manuscript preparation, CR performed statistical analyses to identify genes that showed differentially alterations between scrapie strains and helped with manuscript preparation; AH helped develop experimental plan, authorized and provided lab space and equipment needed for these studies, and helped with manuscript preparation.

## Supplementary Material

Additional File 1 Table showing genes that showed significant expression alterations in scrapie brains at 146 dpi (q-value 5% or less). Click here for file

Additional File 2 **Eisen's Cluster and TreeView representation of genes that showed significant alterations in expression and at least a 1.5 fold change**. For each time point and strain, two scrapie infected mice were compared to each of two mock infected mice, with four hybridizations for each strain at each timepoint, totaling 24 hybridizations. The values from the high powered scan (H) and low powered scan (L), as well as the values from the replicate spots are presented. In the figure, genes are represented in rows and individual hybridizations in the columns. Gene names are indicated for known genes. The hybridization results from the 104 dpi are on the left side of the figure and the hybridization results from the 146 dpi are presented on the right side of the figure. Each colored square indicates the results for a single spot on a hybridized microarray. The spot ratio scrapie:mock is indicated for each of the genes. Red spots indicate increased gene expression in the scrapie sample, green spots indicate a decreased gene expression in the scrapie sample and black spots indicate no change in gene expression. The brightness of the red or green color reflects the relative fold change, with increased brightness correlating with increased fold change. The software clustered the upregulated genes together and the downregulated genes together. Click here for file

## References

[B1] Chesebro B (2003). Introduction to the transmissible spongiform encephalopathies or prion diseases. Br Med Bull.

[B2] Budka H, Aguzzi A, Brown P, Brucher JM, Bugiani O, Gullotta F, Haltia M, Hauw JJ, Ironside JW, Jellinger K (1995). Neuropathological diagnostic criteria for Creutzfeldt-Jakob disease (CJD) and other human spongiform encephalopathies (prion diseases). Brain Pathol.

[B3] Wells GA (1993). Pathology of nonhuman spongiform encephalopathies: variations and their implications for pathogenesis. Dev Biol Stand.

[B4] Prusiner SB (1982). Novel proteinaceous infectious particles cause scrapie. Science.

[B5] McKinley MP, Bolton DC, Prusiner SB (1983). A protease-resistant protein is a structural component of the scrapie prion. Cell.

[B6] Aguzzi A, Polymenidou M (2004). Mammalian prion biology: one century of evolving concepts. Cell.

[B7] Weissmann C (2004). The state of the prion. Nat Rev Microbiol.

[B8] Brown AR, Webb J, Rebus S, Williams A, Fazakerley JK (2004). Identification of up-regulated genes by array analysis in scrapie-infected mouse brains. Neuropathol Appl Neurobiol.

[B9] Booth S, Bowman C, Baumgartner R, Sorensen G, Robertson C, Coulthart M, Phillipson C, Somorjai RL (2004). Identification of central nervous system genes involved in the host response to the scrapie agent during preclinical and clinical infection. J Gen Virol.

[B10] Booth S, Bowman C, Baumgartner R, Dolenko B, Sorensen G, Robertson C, Coulthart M, Phillipson C, Somorjai R (2004). Molecular classification of scrapie strains in mice using gene expression profiling. Biochem Biophys Res Commun.

[B11] Xiang W, Windl O, Wunsch G, Dugas M, Kohlmann A, Dierkes N, Westner IM, Kretzschmar HA (2004). Identification of differentially expressed genes in scrapie-infected mouse brains by using global gene expression technology. J Virol.

[B12] Riemer C, Neidhold S, Burwinkel M, Schwarz A, Schultz J, Kratzschmar J, Monning U, Baier M (2004). Gene expression profiling of scrapie-infected brain tissue. Biochem Biophys Res Commun.

[B13] Zhang Y, Spiess E, Groschup MH, Burkle A (2003). Up-regulation of cathepsin B and cathepsin L activities in scrapie-infected mouse Neuro2a cells. J Gen Virol.

[B14] Baker CA, Martin D, Manuelidis L (2002). Microglia from Creutzfeldt-Jakob disease-infected brains are infectious and show specific mRNA activation profiles. J Virol.

[B15] Baker CA, Manuelidis L (2003). Unique inflammatory RNA profiles of microglia in Creutzfeldt-Jakob disease. Proc Natl Acad Sci U S A.

[B16] Siso S, Puig B, Varea R, Vidal E, Acin C, Prinz M, Montrasio F, Badiola J, Aguzzi A, Pumarola M, Ferrer I (2002). Abnormal synaptic protein expression and cell death in murine scrapie. Acta Neuropathol (Berl).

[B17] Dandoy-Dron F, Guillo F, Benboudjema L, Deslys JP, Lasmezas C, Dormont D, Tovey MG, Dron M (1998). Gene expression in scrapie. Cloning of a new scrapie-responsive gene and the identification of increased levels of seven other mRNA transcripts. J Biol Chem.

[B18] Diedrich JF, Minnigan H, Carp RI, Whitaker JN, Race R, Frey W, Haase AT (1991). Neuropathological changes in scrapie and Alzheimer's disease are associated with increased expression of apolipoprotein E and cathepsin D in astrocytes. J Virol.

[B19] Riemer C, Queck I, Simon D, Kurth R, Baier M (2000). Identification of upregulated genes in scrapie-infected brain tissue. J Virol.

[B20] Diedrich JF, Carp RI, Haase AT (1993). Increased expression of heat shock protein, transferrin, and beta 2- microglobulin in astrocytes during scrapie. Microb Pathog.

[B21] Doh-ura K, Perryman S, Race R, Chesebro B (1995). Identification of differentially expressed genes in scrapie-infected mouse neuroblastoma cells. Microb Pathog.

[B22] Kenward N, Hope J, Landon M, Mayer RJ (1994). Expression of polyubiquitin and heat-shock protein 70 genes increases in the later stages of disease progression in scrapie-infected mouse brain. J Neurochem.

[B23] Duguid JR, Bohmont CW, Liu NG, Tourtellotte WW (1989). Changes in brain gene expression shared by scrapie and Alzheimer disease. Proc Natl Acad Sci U S A.

[B24] Duguid JR, Dinauer MC (1990). Library subtraction of in vitro cDNA libraries to identify differentially expressed genes in scrapie infection. Nucleic Acids Res.

[B25] Diedrich J, Wietgrefe S, Zupancic M, Staskus K, Retzel E, Haase AT, Race R (1987). The molecular pathogenesis of astrogliosis in scrapie and Alzheimer's disease. Microb Pathog.

[B26] Dormont D, Delpech B, Delpech A, Courcel MN, Viret J, Markovits P, Court L (1981). [Hyperproduction of glial fibrillary acidic protein (GFA) during development of experimental scrapie in mice]. C R Seances Acad Sci III.

[B27] Duguid JR, Rohwer RG, Seed B (1988). Isolation of cDNAs of scrapie-modulated RNAs by subtractive hybridization of a cDNA library. Proc Natl Acad Sci U S A.

[B28] Sasaki K, Doh-ura K, Ironside JW, Iwaki T (2002). Increased clusterin (apolipoprotein J) expression in human and mouse brains infected with transmissible spongiform encephalopathies. Acta Neuropathol (Berl).

[B29] Dickinson AG (1976). Scrapie in sheep and goats. Front Biol.

[B30] Dennis GJ, Sherman BT, Hosack DA, Yang J, Gao W, Lane HC, Lempicki RA (2003). DAVID: Database for Annotation, Visualization, and Integrated Discovery. Genome Biol.

[B31] Kascsak RJ, Rubenstein R, Carp RI (1991). Evidence for biological and structural diversity among scrapie strains. Curr Top Microbiol Immunol.

[B32] Bruce ME, Fraser H (1991). Scrapie strain variation and its implications. Curr Top Microbiol Immunol.

[B33] Klein MA, Kaeser PS, Schwarz P, Weyd H, Xenarios I, Zinkernagel RM, Carroll MC, Verbeek JS, Botto M, Walport MJ, Molina H, Kalinke U, Acha-Orbea H, Aguzzi A (2001). Complement facilitates early prion pathogenesis. Nat Med.

[B34] Caughey B, Raymond GJ, Ernst D, Race RE (1991). N-terminal truncation of the scrapie-associated form of PrP by lysosomal protease(s): implications regarding the site of conversion of PrP to the protease-resistant state. J Virol.

[B35] Caughey B, Raymond GJ (1991). The scrapie-associated form of PrP is made from a cell surface precursor that is both protease- and phospholipase-sensitive. J Biol Chem.

[B36] McKinley MP, Taraboulos A, Kenaga L, Serban D, Stieber A, DeArmond SJ, Prusiner SB, Gonatas N (1991). Ultrastructural localization of scrapie prion proteins in cytoplasmic vesicles of infected cultured cells. Lab Invest.

[B37] Fevrier B, Vilette D, Archer F, Loew D, Faigle W, Vidal M, Laude H, Raposo G (2004). Cells release prions in association with exosomes. Proc Natl Acad Sci U S A.

[B38] Zlotnik I, Rennie JC (1963). Further observations on the experimental transmission of scrapie from sheep and goats to laboratory mice. J Comp Pathol.

[B39] Chandler RL (1963). Res Vet Sci.

[B40] BMAP cDNA libraries and clones derived from brain regions of adult mouse (strain C57BL6) were obtained from Research Genetics, Inc. These clones and libraries were constructed by M. Bento Soares, Ph.D. at the University of Iowa under contract NO1 MH80014, which was awarded by the National Institute of Mental Health (NIMH) and the National Institute of Neurological Disorders and Stroke (NINDS) as part of the Brain Molecular Anatomy Project (BMAP) . BMAP is an NIH initiative to support molecular cartography of the mammalian nervous system through the localization of genes and the analysis of their expression patterns.. http://wwwresgencom/products/BMAPphp3.

[B41] Tanaka TS, Jaradat SA, Lim MK, Kargul GJ, Wang X, Grahovac MJ, Pantano S, Sano Y, Piao Y, Nagaraja R, Doi H, Wood WH, Becker KG, Ko MS (2000). Genome-wide expression profiling of mid-gestation placenta and embryo using a 15,000 mouse developmental cDNA microarray. Proc Natl Acad Sci U S A.

[B42] Tusher VG, Tibshirani R, Chu G (2001). Significance analysis of microarrays applied to the ionizing radiation response. Proc Natl Acad Sci U S A.

[B43] Storey (2002). A direct approach to false discovery rates. J Roy Stat Soc Ser B.

[B44] Rice J (1995). Mathematical Statistics and Data Analysis.

[B45] Eisen MB, Spellman PT, Brown PO, Botstein D (1998). Cluster analysis and display of genome-wide expression patterns. Proc Natl Acad Sci U S A.

[B46] Diehn M, Sherlock G, Binkley G, Jin H, Matese JC, Hernandez-Boussard T, Rees CA, Cherry JM, Botstein D, Brown PO, Alizadeh AA (2003). SOURCE: a unified genomic resource of functional annotations, ontologies, and gene expression data. Nucleic Acids Res.

[B47] Livak KJ, Schmittgen TD (2001). Analysis of relative gene expression data using real-time quantitative PCR and the 2(-Delta Delta C(T)) Method. Methods.

[B48] Tatusova TA, Madden TL (1999). BLAST 2 Sequences, a new tool for comparing protein and nucleotide sequences. FEMS Microbiol Lett.

