# Supplementary figures and images for: Gene expression alterations in brains of mice infected with three strains of scrapie
Source: BMC Genomics. 2006 May 16;7:114. doi: 10.1186/1471-2164-7-114 (PMC1475852; doi:10.1186/1471-2164-7-114)

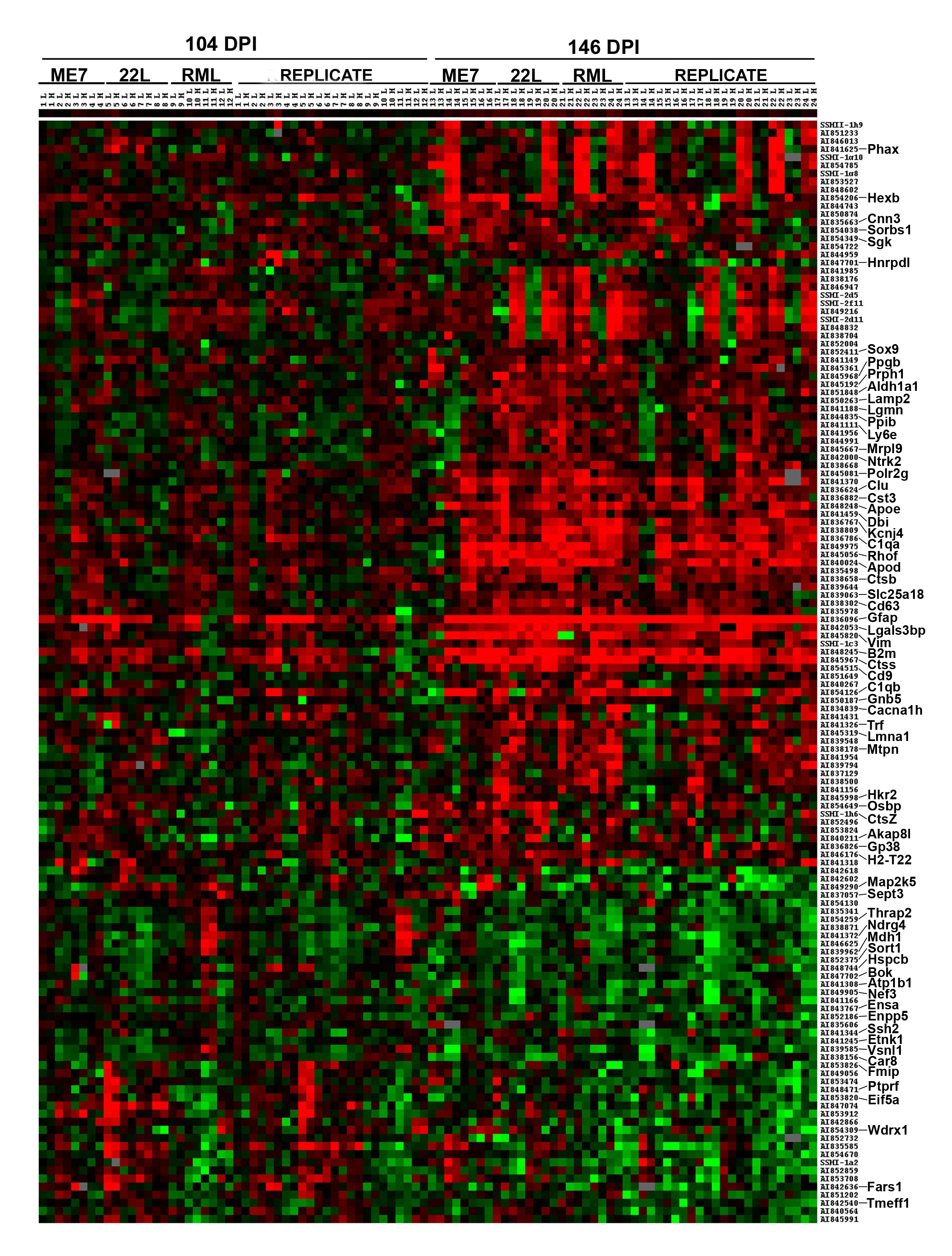

Supplement: Additional File 2 — Eisen's Cluster and TreeView representation of genes that showed significant alterations in expression and at least a 1.5 fold change. For each time point and strain, two scrapie infected mice were compared to each of two mock infected mice, with four hybridizations for each strain at each timepoint, totaling 24 hybridizations. The values from the high powered scan (H) and low powered scan (L), as well as the values from the replicate spots are presented. In the figure, genes are represented in rows and individual hybridizations in the columns. Gene names are indicated for known genes. The hybridization results from the 104 dpi are on the left side of the figure and the hybridization results from the 146 dpi are presented on the right side of the figure. Each colored square indicates the results for a single spot on a hybridized microarray. The spot ratio scrapie:mock is indicated for each of the genes. Red spots indicate increased gene expression in the scrapie sample, green spots indicate a decreased gene expression in the scrapie sample and black spots indicate no change in gene expression. The brightness of the red or green color reflects the relative fold change, with increased brightness correlating with increased fold change. The software clustered the upregulated genes together and the downregulated genes together. [file 1471-2164-7-114-S2.png]
